# Mitochondrial Control in Inflammatory Gastrointestinal Diseases

**DOI:** 10.3390/ijms232314890

**Published:** 2022-11-28

**Authors:** Guo-Yan Sui, Feng Wang, Jin Lee, Yoon Seok Roh

**Affiliations:** 1 College of Pharmacy and Medical Research Center, Chungbuk National University, Cheongju 28160, Republic of Korea; suiguoyan1996@gmail.com (G.-Y.S.); wang979253414@gmail.com (F.W.); 2 Department of Pathology, School of Medicine, University of California, San Diego, La Jolla, CA 92093, USA

**Keywords:** mitochondria, biogenesis, mitophagy, inflammatory bowel disease, colorectal cancer

## Abstract

Mitochondria play a central role in the pathophysiology of inflammatory bowel disease (IBD) and colorectal cancer (CRC). The maintenance of mitochondrial function is necessary for a stable immune system. Mitochondrial dysfunction in the gastrointestinal system leads to the excessive activation of multiple inflammatory signaling pathways, leading to IBD and increased severity of CRC. In this review, we focus on the mitochondria and inflammatory signaling pathways and its related gastrointestinal diseases.

## 1. Mitochondria-Mediated Inflammation

Mitochondria are integral to the maintenance of normal cellular functions because they are responsible for eukaryotic energy production, phospholipid and heme synthesis, calcium homeostasis, apoptosis activation, and cell death [1,2]. Mitochondria have a genome (mtDNA) that replicates independently of the host genome [3]. Defects in mitochondrial DNA (mtDNA) can lead to various diseases [4]. Mitochondria are membrane-bound organelles found in almost all eukaryotic cells; they consist of inner and outer membranes, which separate and maintain the aqueous compartment, intermembrane space, and matrix [5]. Mitochondria contribute to many processes critical to cellular function and dysfunction, and their shape and localization in the cell are critical and tightly regulated by fission and fusion, biogenesis, and autophagy processes, ensuring a relatively stable mitochondrial population [6,7,8,9]. Multiple lines of evidence suggest that mitochondria are the primary regulators of inflammation. Mitochondrial dysfunction triggers various immune responses and accelerates inflammation and tumor progression [10]. Overall, mitochondrial homeostasis is essential for the healthy development of living organisms [6].

### 1.1. Mitochondrial Dynamics and Inflammation

Mitochondria are generally described as isolated organelles [11], and studies have shown that they maintain normal cellular metabolism through frequent fusion and division [6,12,13]. Mitochondrial dynamics determine mitochondrial morphology and are influential for cellular function [14,15]. Research on mitochondria has found that the homeostasis of fusion and division is the basis for organisms to accomplish various physiological and pathological repair processes [16,17]. Simultaneously, mitochondria can remove damaged and dysfunctional mitochondria through cyclic fission and fusion, achieving quality control [8,18,19]. The dysfunction of mitochondrial fusion and fission leads to mitochondrial damage, activating inflammatory responses through different pathways.

#### 1.1.1. Mitochondrial Fusion

Mitochondrial fusion promotes oxidative phosphorylation (OXPHOS) and redistribute mtDNA between damaged and healthy mitochondria [20]. The mitochondrial fusion process is divided into two main categories: fusion of the inner (IMM) and outer mitochondrial membranes (OMM) and fusion between mitochondria, which are coordinated with each other almost simultaneously [21]. During mitochondrial fusion, there are two kinetically relevant inducible proteins, optic atrophy1 (OPA1) and mitochondrial fusion protein 1/2 (MFN1/2) [22,23]. Endosomal fusion is mediated by OPA1, located in the IMM [24]. However, OPA1 deletion not only weakens mitochondrial fusion but also leads to mitochondrial breakage [25]. MFN1/2 mediates mitochondrial outer membrane fusion, and the two proteins are structurally and functionally similar [26]. The molecular mechanisms of mitochondrial fusion are highly regulated. The absence of intracellular MFN1 or MFN2 leads to mitochondrial fragmentation into granules, whereas the absence of fusion function leads to an increase in the severity of severe mitochondrial fragmentation and severe impairment of mitochondrial function [27]. Additionally, Mfn2 has physiological functions, such as energy production regulation, mitochondrial endoplasmic reticulum coupling, and autophagy [28,29]. Mitochondrial fusion promotes the exchange of mitochondrial substances, including proteins, lipids, and other small molecules [20]. This process helps maintain homeostasis of the mitochondrial network structure, enhance endoplasmic reticulum coupling, and optimize the expression of mitochondrial functions to avoid the continuously damage accumulation induced by mtDNA mutations [18]. Impaired mitochondrial fusion leads to mitochondrial dysfunction, leading to a deficiency in OXPHOS, depletion of mtDNA, production of reactive oxygen species (ROS). Those response will activate early inflammatory mediators, including tumor necrosis factor α (TNF-α), interleukins, interferon-γ (IFN-γ), and reactive oxygen species (RNS), and various inflammatory signaling pathways, exacerbating the progression of inflammatory diseases.

#### 1.1.2. Mitochondrial Fission

**Figure 1:**
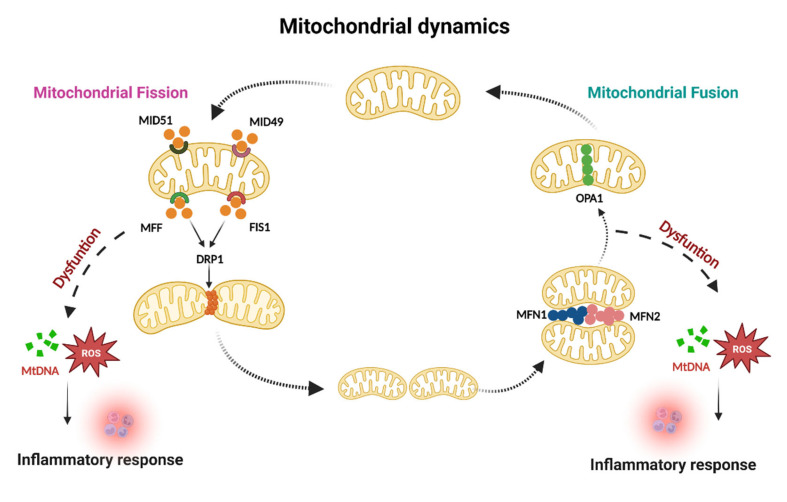
Mitochondrial dynamics and inflammation.

Mitochondrial division refers to the overlap of the mitotic opening site with the coupling site of the mitochondrial endoplasmic reticulum structure [30], accumulation of associated proteins that mediate mitosis, and eventual severing of the parent mitochondrion to form two suborganelles [31]. The mitochondrial division promotes cell proliferation, ROS production, and mitophagy [32,33]. In mammalian cells, mitochondrial dynamics proteins of 49 and 51 kDa (MID49/51), fission protein 1 (FIS1), mitochondrial fission factor (MFF), and dynamin-related protein 1 (DRP1) are major regulators of the mammalian mitochondrial division [34]. The DRP1 protein has a significantly different mode of action from the OPA1 protein and MFN1/2 in that it must bind to a specific receptor to mediate mitochondrial division, and the associated outer membrane protein and DRP1 receptor must be present within the outer membrane of the mitochondria [35,36]. MFF, FIS1, and MID49/51 act as DRP1 receptors [37]. Studies have shown that DRP1 overexpression accelerates mitochondrial division and leads to massive mitochondrial fragmentation [38]. Conversely, knocking out DRP1 or MFF inhibits mitochondrial division [39]. Similar to DRP1, MFF, FIS1, and MID49/51 are associated with mitochondrial division. MFF is a tail membrane protein embedded in the outer membrane, which not only functions as a mitochondrial binding site but also acts as a translocation site for DRP1 to the OMM, activating DRP1 to induce mitochondrial division and regulating division [40]. By contrast, Mdivi-1 is a mitochondrial division inhibitor that rapidly and reversibly inhibits the formation of network mitochondria by preventing DRP1 self-assembly and inhibiting GTPase activity [41]. Disruption of mitochondrial fission induces mitophagy defect, and damaged mitochondria cannot perform normal metabolism, creating a large accumulation of ROS and mtDNA, thereby causing to the development of several chronic inflammatory diseases. In conclusion, mitochondrial dynamics are a critical way to regulate mitochondrial function and the subsequent inflammatory response as shown Figure 1.

Mitochondrial fission and fusion are essential for maintaining mitochondrial homeostasis and subsequent inflammatory response. Mitochondrial fission promotes the removal of damaged or senescent mitochondria, MID49, FIS1, MID51, and MFF have been identified as the major regulators for mitochondrial fission and the DRP1 receptors. The coordinated interaction of these proteins is important for normal mitochondrial division. Dysfunction of mitochondrial fission will release mtDNA and promote ROS production to activate an inflammatory response further. In addition, mitochondrial fusion allows mtDNA redistribution among the damaged and healthy mitochondria to maintain mitochondrial homeostasis by two kinetically relevant inducible proteins known: OPA1 and MFN1/2. Defective mitochondrial fusion results in mitochondrial dysfunction, which further causes mtDNA depletion and ROS production, exacerbating the inflammatory response.

### 1.2. Mitophagy and Inflammation

The core function of mitochondria is energy generation for maintaining tissue homeostasis and conduits for programmed apoptosis requires strict control the quality and quantity of mitochondria [8,42]. Disturbances in mitochondrial homeostasis can lead to cell death, leading to a range of metabolic disorders [43]. Mitophagy uses lysosomes to selectively remove dysfunctional or damaged mitochondria, maintaining mitochondrial quality [44]. In addition, degraded components can be used as raw materials for the synthesis of biomolecules [45]. Mitophagy defect causes to excessive damaged mitochondria accumulation, and damaged mitochondrial components and metabolites are released into the cytoplasmic or extracellular environment as damage-associated molecular patterns (DAMP) to induce inflammation.

#### 1.2.1. Parkin/Pink-Mediated Mitophagy

**Figure 2:**
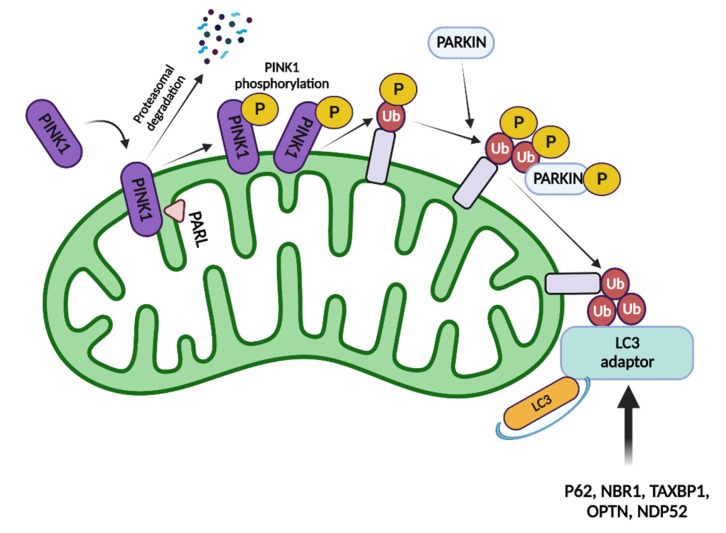
LC3 adaptor recognizes ubiquitinated mitochondrial proteins and induces mitophagy via ubiquitin (Ub)-dependent mechanisms.

Parkin- and PINK-mediated mitophagy occurs mainly during the quality control of damaged mitochondria [46]. Studies have found that mutations in Parkin or PINK caused mitochondrial lesions and were associated with mitochondrial fusion and division processes in Drosophila [47]. Follow-up studies have shown that ubiquitination is an important critical marker for mitochondrial damage [48], and PINK1, a kinase of ubiquitin, phosphorylates ubiquitin Ser65, which can promote the activation of Parkin (E3 ligase of ubiquitination reaction) only after phosphorylation [49]. Mitochondrial outer membrane proteins (e.g., VDAC1) labeled by ubiquitination can recruit the mitophagy receptor P62/SQSTM1 and promote the binding of damaged mitochondria to autophagic vesicles [50]. Ubiquitin phosphorylation can also directly recruit autophagy receptors, such as optineurin (OPTN) and nuclear dot protein 52 (NDP52), to mitochondria and induce mitophagy [50]. The deubiquitinating enzyme ubiquitin-specific protease 30 can de-flag ubiquitinated mitochondria and reduce mitophagy [51]. PINK1 is continuously expressed in mitochondria and transported into mitochondria by the membrane transport enzymes TOM and TIM complexes, after which the N-terminus of PINK1 is degraded by the protease PARL, while its C-terminus is transported out of mitochondria for degradation by the proteasome [52,53]. PINK1 can also be degraded in the mitochondrial matrix, such that PINK1 remains at a relatively low level in normal cells [54]. Damage to mitochondria may lead to mitochondrial depolarization, leading to the inability of PINK1 to enter the mitochondria for degradation [55]. PINK1 is activated by autophosphorylation and phosphorylates the serine residue at position 65 of a nearby ubiquitin molecule [56]. Phosphorylated ubiquitin molecules bind and recruit Parkin molecules, and PINK1, in turn, phosphorylates Parkin and activates it. Activated Parkin can polyubiquitinate various mitochondrial protein substrates [57]. In the presence of LC3 splice proteins, autophagosomes target mitochondria and induce mitophagy as shown Figure 2.

There are five main LC3 junction proteins: P62, OPTN, NDP52, BRCA1 gene protein (NBR1), and TAX1 binding protein 1 (TAX1BP1) [58]. LC3 splice proteins must have two structural domains: the ubiquitin-binding domain (UBD) and the LC3-interacting region (LIR). They bind polyubiquitinated substrates coupled to the mitochondrial protein K63 through the UBD domain and recognize and bind LC3 on the autophagosome membrane through its LIR, using the junction protein as a bridge to target autophagosomes to mitochondria tagged with ubiquitin molecules and induce mitophagy [59]. The recruitment of OPTN to mitochondria activates TANK-binding kinase 1 (TBK1), which further phosphorylates OPTN and increases mitophagy [60]. Although other junctional proteins can also be phosphorylated by TBK1, OPTN and NDP52 are the most common substrates of TBK1 [61].

Under normal cellular physiology, PINK is translocated into IMM and OMM by the action of translocases. PARL cleaves PINK1 and PINK1 was translocated to the cytosol for rapid degradation. When mitochondria are damaged, the mitochondrial membrane potential is depolarized and PINK1 accumulates in the outer mitochondrial membrane in response to TOM and is not degraded. PINK1 is activated by autophosphorylation and phosphorylates the serine residue at position 65 of the ubiquitin molecule. The phosphorylated ubiquitin molecule recruits and binds PARKIN, which in turn phosphorylates PARKIN to activate it. Activated PARKIN modifies mitochondrial protein substrates with polyubiquitination. In the presence of LC3 junction protein, autophagosomes are targeted to mitochondria, inducing mitophagy to occur.

#### 1.2.2. Nix-Mediated Mitophagy

Nix-mediated mitophagy plays a major role in erythrocytes [62]. In most mammals, erythrocytes do not have mitochondria, and mitochondrial clearance is mainly accomplished by mitophagy during reticulocyte maturation [63]. In Nix-deficient mice, mitochondria remain present in mature erythrocytes, and clearance of mitochondria by Nix is regulated by zinc finger proteins (KRAB-ZFPs) containing the KRAP structural domain [64]. Nix contains a BH3 structural domain that interacts with the Bcl-2 and Bcl-xL [65]. A specific inhibitor (ABT-263) of Bcl-2 and Bcl-xL, which can induce mitophagy in Nix-deficient reticulocytes, suggesting that the Bcl-2 protein family plays a regulatory role in mitophagy [66]. Although mitophagy does not require nutrient deficiency to induce the initiation complex in autophagy, Unc-51-like kinase 1, a non-selective pre-autophagy initiation complex, is reportedly involved in mitophagy in reticulocytes [67]. Nix can also mediate mitophagy during stem cell differentiation, indicating that Nix might play a role not only in mitochondrial clearance during erythroid maturation but also in other developmental processes [64].

#### 1.2.3. Mitophagy Dysfunction-Mediated Inflammation

Mitophagy is essential for the mitochondrial quality and homeostasis [6]. Impaired mitophagy is associated with the pathogenesis of inflammatory diseases [68]. Evidence suggests that Parkin and PINK deficiency causes impaired mitophagy and further induces inflammatory response by mtDNA release [69]. In addition, Parkin and PINK alleviated STING-mediated inflammation, indicating the role for PINK1 and Parkin-mediated mitophagy in suppressing innate immunity [70]. In addition to exogenous DAMP-induced inflammation, mitochondrial homeostasis in macrophages is critical for their activation. Mitophagy is activated in the process of M1 macrophage polarization in response to LPS/IFNγ treatment, accelerating metabolic reconnection to glycolysis [71]. Notably, NIX-depleted M1 macrophages have reduced glycolytic enzymes levels and pro-inflammatory cytokines, suggesting that they are metabolically defective during differentiation [72]. In addition, ISG15-deficient macrophages exhibit decreased levels of Parkin and defective mitophagy is response to IFNγ stimulation. Deletion of ISG15 causes defective macrophage polarization, resulting in improved viral susceptibility [73]. Overall, both the exogenous impaired mitophagy release of DAMP and defective autophagy by macrophages are important for the inflammatory regulation.

### 1.3. Mitochondria and ROS

As highly dynamic organelles, mitochondria are crucial for cell physiology. This is the main site of not only intracellular OXPHOS and synthesis of adenosine triphosphate (ATP) but also ROS production, which is closely linked to energy metabolism disorders [17,74]. ROS are extremely reactive oxygen-containing molecules generated by the incomplete reduction of oxygen under normal and pathological conditions or upon exposure to environmental or exogenous chemicals; they stimulate relevant signaling pathways in cells upon changes in the intracellular and extracellular environment [75,76].

A decrease or increase in ROS levels may generate dangerous signals which stimulates specific redox-sensitive signaling pathways. Once activated, these different signals may have disruptive or protective roles. Within the normal fluctuations of energy load, ROS production and levels are beneficial for maintaining the normal physiological functions of specific biological systems in mitochondria, cells, and tissues [77]. For example, ROS may act as secondary messengers in response to changes in growth factors, hormones, cytokines, and intra- and extracellular ATP [78]. In addition, macrophages and neutrophils containing NADPH oxidase can protect themselves from foreign microorganisms via ROS production [79,80]. Although ROS plays an essential role in cell signaling, defense against microbial invasion, and some key metabolic pathways, they also have toxic and damaging effects. When ROS production exceeds the neutralizing capacity of endogenous enzymes and non-enzymatic antioxidant systems, many physiological or pathological states of the body stimulate further increases in ROS levels, leading to oxidative stress [81].

As the main site of OXPHOS in cells, mitochondria plays a key role in energy metabolism. Several flat organisms require exogenous food or nutrients to synthesize ATP, most of which is generated by mitochondrial OXPHOS [82]. The superoxide anion, the main component of ROS, is closely associated with ATP production [83]. During energy metabolism, NADH and FADH_2_, as hydrogen transporters, can transfer electrons captured from the tricarboxylic acid cycle to the mitochondrial respiratory chain (MRC), where most electrons migrate along the MRC to cytochrome oxidase and later combine with oxygen molecules to form water [84]. However, a small fraction of electrons (approximately 2%) from complexes I and III leaks out from the MRC and reacts with oxygen molecules to produce superoxide anions, which can be converted to H_2_O_2_ spontaneously or under the effect of manganese superoxide dismutase [85]. In addition, superoxide anions react with nitrous oxide to form peroxynitrite. Hydroxyl radicals and peroxynitrite are high reactivity and can make mitochondrial membranes damage, proteins, and mtDNA, damaging mitochondrial function and the genome [76].

Apoptosis refers to the active and orderly process of cell death through gene regulation to maintain the growth and homeostasis of the organism with a number of changes of morphology and biochemistry [86]. Pathways that mediate apoptosis are mainly divided into endogenous and exogenous mitochondrial pathways.

In the endogenous pathway, MOM permeabilization leads to cytochrome C release into the cytoplasm, regulated by Bcl-2 family proteins [87]. Bax and Bak belong to Bcl-2 family, which are activated and bind to the OMM, forming pores in the mitochondria that lead to the cytoplasm allowing proteins inside mitochondria to pass through the mitochondrial membrane [88]. The cytochrome C molecule has a binding site for Apaf-1, which is activated by ATP when cytochrome C in the cytoplasm binds to the bridging protein Apaf-1 [89]. Activated Apaf-1 activates caspase-9, forming apoptotic vesicles composed of caspase-9, Apaf-1, and cytochrome C. This apoptotic vesicle has a cleavage effect on apoptotic protease caspase-7, which is activated by cleavage and degrades downstream proteins related to cell life, leading to apoptosis [90]. In addition to cytochrome C-mediated apoptosis, another apoptotic pathway exists in the mitochondrial pathway that does not depend on classical caspases, namely, apoptosis-inducing factor (AIF) protein-mediated apoptosis. AIF, normally located inside mitochondria, is released from mitochondria into the cytoplasm and eventually into the nucleus, causing DNA damage and cell death [91].

**Figure 3:**
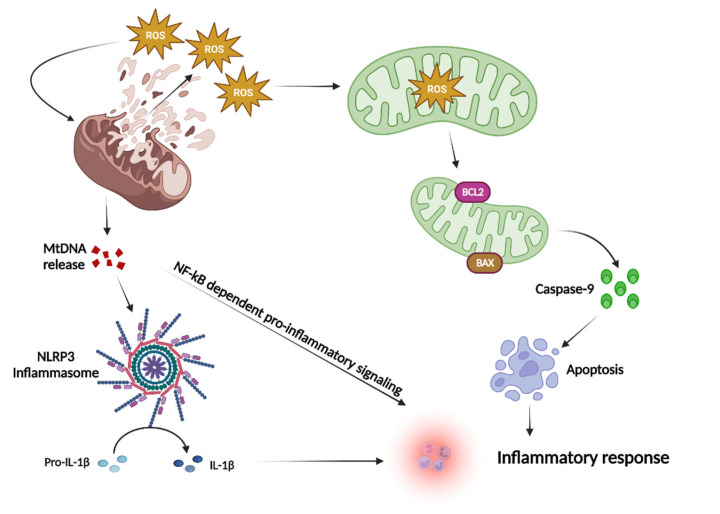
mtROS plays multiple roles in inflammatory responses.

Mitochondrial ROS (mtROS) plays multiple roles in inflammatory responses as shown in Figure 3. First, the massive production of ROS causes severe damage to the mitochondrial membrane, accompanied by the onset of lipid peroxidation and changes in mtDNA, further exacerbating mitochondrial dysfunction and ROS production. MtROS accumulation activates NFKB and the inflammatory factors expression. In addition, mtROS and mtDNA recruit pro-caspase-1, process pro-IL-1, and pro-IL-18 to promote cytokine release, mediating activation of the inflammatory vesicle NLRP3 and NLRC4 complexes. Finally, the release of mtROS into the extracellular environment further exacerbates the inflammatory response.

Mitochondria, as the primary site of ROS sources, are essential for maintaining ROS levels and inflammatory responses. Damaged mitochondria release large amounts of ROS, while the massive production of ROS further exacerbates mitochondrial damage, accompanied by the massive release of mtDNA and mtROS. First, the enormous accumulation of mtROS directly induces the inflammatory response through activation of NFkB and inflammatory factor expression. Second, mitochondrial release of DNA recruits caspase-1, processes pro-IL-1, and pro-IL-18, and releases pro-inflammatory factors that mediate the NLRP3 activation and NLRC4 inflammasome complexes. Finally, mtROS are released into the extracellular environment, further exacerbating the inflammatory response. In addition. Excessive ROS production induces mitochondrial-mediated apoptosis. ROS leads to mitochondrial membrane depolarization and Bax/Bcl2 channel opening on the OMM, which releases cytochrome c into the cytoplasm. cytochrome c forms an apoptotic vesicle complex with procaspase-9 in the cytoplasmic lysate, leading to caspase-9 activation. Caspase-9 activates effector cystathionine, leading to cellular protein cleavage and apoptosis leading to cell death and thus mediating the inflammatory response.

## 2. Gastrointestinal System Pathophysiology

The gastrointestinal tract is the largest immune and detoxification organ [92]. The basic function of the gastrointestinal tract is the food digestion and absorption, providing the body with the substances and energy it requires. Many organs are involved in the digestive system, including the esophagus, stomach, liver, gallbladder, and intestinal tract, which comprise the digestive tract. Recently, gastrointestinal diseases prevalence has increased annually, becoming one of the major diseases affecting human health [93]. Gastrointestinal diseases are divided into several major categories: IBD, intestinal epithelial barrier dysfunction, and CRC [94,95,96].

### 2.1. Inflammatory Bowel Diseases

IBD is a specific inflammatory disease involving ileum and colorectum, and its pathology consists mainly of inflammatory infiltration of the intestinal epithelium and necrosis [97]. History of IBD increases the risk of CRC and accounts for approximately 2% of all CRC cases. In Europe, 2.5 million individuals have IBD, with new cases reported every year, and with the high cost of health care [98]. IBD consists mainly of ulcerative colitis (UC) and Crohn’s disease (CD) [99]. UC is a non-specific inflammatory and chronic disease of the intestine, characterized by varying degrees of systemic symptoms, and continuous and diffuse inflammatory changes in the colonic mucosa [100]. In recent years, because of the increase in the incidence of UC and the risk of CRC, this has been listed by the World Health Organization as a modern intractable disease [101]. CD is non-specific enteritis characterized by chronic granulomatous inflammatory lesions associated with autoimmune deficiency, infection, and genetic factors [102]. The prevalence of CD is high in developed countries, such as North America and Western Europe. CD can involve the entire gastrointestinal tract, with a whole-mural inflammation and segmental distribution, andorgans outside the gastrointestinal tract, such as joints, skin, eyes, liver, gallbladder, and pancreas [103].

IBD etiology and pathogenesis have not been fully investigated and are presumably related to immune abnormalities, genetics, infection, the environment, and psychiatric factors [104]. The literature has suggested that first-degree relatives of patients with IBD are at high risk of IBD development and that the prevalence is higher in identical twins than in heterozygotic twins [105]. Thus, genetic studies have consistently concluded that genetic factors have a greater impact on CD than on UC. The exact mechanisms by which these factors trigger IBD or contribute to IBD recurrence remain unclear [106]. Epidemiological surveys have shown that dietary factors are one of the main reasons for regional variability in the incidence of IBD. Evidences suggested that a high animal fat intake, eggs, and dairy products may increase IBD prevalence, possibly because of the dietary structure of the intestinal mucosa, intestinal immunity, and intestinal microenvironment [107]. The high salt concentrations in processed foods can upregulate the pro-inflammatory cytokines expression, affecting immune and inflammatory responses in the gut [108]. The literature has also shown that the function of vitamin D is diverse and associated with various diseases, including IBD. In mouse models, vitamin D deficiency and sodium dextran sulfate induce increased susceptibility to colitis in intestinal pathology [109].

### 2.2. Colorectal Cancer

Among colorectal malignancies, the most common is CRC, a collective term for colon and rectal cancer [110]. IBD is one of the causes of CRC, however, the primary cause is adenomatous polyps [111]. According to the World Health Organization, approximately 1.9 million individuals per year are diagnosed with colorectal cancer, of which 70% of cases are of colon cancer and 30% of rectal cancer and the number of deaths per year due to CRC is at least 0.9 million [112]. CRC often has no specific symptoms in its early stages and is thus often overlooked and missed. The earliest stages of CRC may only show symptoms such as abdominal distension, abdominal discomfort, and indigestion. As the disease progresses, symptoms such as an increased frequency of bowel movements, diarrhea, and constipation may be observed. The symptoms of late-stage CRC are related to the location, size, infiltration, invasion, and metastasis of the tumor. The tumor volume increases with tumor growth, leading to anemia and wasting disease manifestations associated with chronic gastrointestinal bleeding [113].

The occurrence and progression of CRC are often associated with genetic factors. People with a history of colorectal cancer from family usually have an high risk of developing cancer owing to genetic factors that cause patients to inherit the same defective genes, with family genetics accounting for approximately 5% of all CRC cases [114]. The development of colon cancer is associated with specific lifestyle habits. Dietary habits, such as high fat diet, and a low-folic acid diet, increase the incidence of CRC; however, high consumption of fruits and vegetables prevents CRC [115]. In addition, animal studies have shown that calcium can bind to fatty acids and bile, reducing their irritation and damage to mucosal cells in the intestine, which might have a protective effect; however, further studies on its role and mechanism are necessary [116]. Insufficient exercise and obesity promote the occurrence of CRC, and the CRC incidence is significantly higher in individuals with an increased body mass index [117].

## 3. Mitochondrial Control of Inflammation in Gastrointestinal System Pathophysiology

### 3.1. Mitochondria and Inflammation in IBD

Evidence have revealed the association between mitochondria and IBD. IBD, a disease caused by energy deficiency in the intestinal epithelium, is closely connected with mitochondrial damage in the intestinal epithelium. IBD-related intestinal inflammation leads to mitochondrial dysfunction [118]. IBD mainly includes UC and CD, inflammatory diseases caused by abnormal intestinal-specific immune regulation that are highly recurrent and difficult to cure [119]. The mechanisms of intestinal mucosal barrier damage and inflammation are mitochondrial-related [118]. Mitochondrial homeostasis is the process of intracellular mitochondrial self-healing after ROS stimulation, cellular senescence, nutritional deficiency, or bacterial or viral infections. Mitochondrial dysfunction can promote inflammation and oxidative stress and induce apoptosis [85].

#### 3.1.1. Mitophagy-Inflammation-IBD

Mitophagy, involved in many autoimmune diseases, is closely associated with IBD [120]. Dysregulation of mitophagy in the intestinal epithelium is a major predisposing factor for immune malfunction and inflammatory processes in the intestine [121]. External stimuli or genetic mutations may be predisposing factors for mitochondrial dysfunction and mitophagy dysregulation, which can affect normal cellular metabolism and lead to increased permeability of the intestinal epithelium [122]. Defective mitophagy leads to the body loss the ability to eliminate or accumulate damaged mitochondria, leading to ROS and mtDNA release, increasing the oxidative stress response, inflammation, and incidence of IBD.

Mitochondrial degradation is closely associated with a series of Atg family proteins, such as Atg32, Atg7, and Atg8, which act on the mitochondrial surface and promote the assembly of core Atg proteins in mitochondria [123,124]. p62/SQSTM1 binds to the phagocytosed mitochondrial membrane surface Atg8 homolog LC3 to induce the formation of autophagic vesicles, inducing mitochondrial breakdown [125]. Evidence has shown that deficiency of the intestinal antigen-presenting cell ATG7 leads to abnormal mitochondrial function and oxidative stress, leading to enhanced immunopathology and inflammatory Th17 responses that exacerbate the susceptibility of dextran sulfate sodium (DSS)-induced colitis [126]. Genome-wide searches have recognized several IBD-related genes of autophagy and mitophagy, such as ATG16L1, immunity-related GTPase M (IRGM), and leucine-rich repeat kinase 2 (LRRK2) [127]. In functional studies of CD-related risk variants of ATG16L1 and IRGM, further impairment of mitophagy was observed, evidenced by altered mitochondrial morphology, decreased mitochondrial membrane potential, increased mtROS, and disrupted LC3-labeled mechanisms [128,129]. According to the evidence, ATG16L1 deficiency resulted in increased ROS production and impaired mitophagy, which exacerbated colitis in a mouse model through increasing Il-1β expression and TNFα inflammatory factors.

Regarding LRRK2, Parkinson’s disease-associated mutation G2019S can promote mitophagy by inducing mitochondrial fission and interacting with ULK1 [130]. Notably, recent cross-phenotyping research found that psoriasis (risk), CD, and UC share the intronic SNP rs3766606 for PARK7. Like LRRK2, PARK7 is a classic PD risk locus and a potential candidate gene for UC [131]. PARK7 plays a clear role in maintain mitochondrial homeostasis and mitophagy and belongs to the PARK family [132]. The PINK1/Parkin plays an essential role in mitophagy regulation, and HSF2 enhances mitophagy in UC, reduces intracellular ROS, and inhibits NLRP3 inflammasome activation by PARL/PINK1/Parkin signaling pathway, alleviating mucosal inflammation [133].

Mitochondrial dysfunction in the intestinal epithelium contribute to IBD pathogenesis by exacerbating inflammatory response. NIX regulates mitophagy and is a critical protein for cellular homeostasis. It binds to LC3, inducing mitophagy. In mouse experiments, mitophagy was impaired in NIX gene-deficient mice, and strong inflammatory features and loss of mucosal integrity [134].

Inhibitor prohibitin (PHB) has multiple functions in cells and is an important protein for maintaining mitochondrial respiration and regulating mitophagy [135]. PHB is mainly located in mitochondria of intestinal epithelial cells. PHB expression is reduced in colon biopsies with IBD than in those of normal tissues [136]. According to the literature, PHB deficiency in intestinal epithelial cells induces mitochondrial dysfunction and defective mitophagy, resulting in increased expression of the pro-inflammatory cytokines IL-1β, TNF-α, and interferon-γ (Ifnγ) in the ileum, along with reduced anti-inflammatory Il-10, exacerbating the inflammatory response [137]. PHB overexpression reduced ROS accumulation and increased oxidative stress-induced permeability in intestinal epithelial cells. In the DSS-induced IBD model, protein carbonyl content increased in wild-type mice but not in PHB transgenic mice during DSS induction, and protein carbonyl content was the most prevalent and sufficient indicator of severe oxidative protein damage, suggesting that PHB overexpression reduces oxidative stress, mitigates mucosal barrier breakdown, and alleviates the inflammatory response by reducing the IL-1β, TNF-α, and IFN-γ mRNA levels induced by DSS in IBD [138].

Maintenance of the mucosal barrier function integrity of intestinal epithelial cells depends on energy supply, and mitochondrial function may be crucial for the protection of the mucosal barrier function of intestinal epithelial cells [139]. Mitochondrial dysfunction leads to damage of intestinal epithelial cells (e.g., Paneth cells and cup cells are dysfunctional or absent), leading to the reduced barrier function and increased permeability of intestinal epithelial cells and stimulating development of intestinal inflammation [122]. Damage and reduction of Paneth cells are important mechanisms for intestinal pathology development in CD [140]. Mitochondrial dysfunction leads to a decrease in mitochondrial endosomal anti-proliferative proteins in Paneth cells, which increases ileal inflammation in patients with CD [141]. Polymorphism of the Atg16L1 is connected with CD, and the intestinal mucosa without Atg16L1 shows Paneth cell deficiency and TNF-mediated cell necrosis, whereas TNF-α or receptor-interacting protein kinase inhibitors reduce inflammation in IBD models [142]. Zhang et al. showed that Atg16L1 deficiency leads to altered macrophage function and exacerbates CD disease [129]. Liu et al. showed that knockdown of the immune-related GTPase family M protein 1 (Irgm1) gene in mice with DSS-induced colitis may regulate the acute inflammatory response in the mouse intestine by regulating mitophagy in intestinal epithelial cells and Paneth cells [128].

#### 3.1.2. ROS-Inflammation-IBD

The main source of ROS is mitochondria. The increasing evidence supported that ROS is involved in inflammatory process and development and that excessive ROS production leads to cell and tissue damage, which triggers the development of IBD [85]. ROS play a central role in gastrointestinal disorders [143]. Prolonged oxidative stress reduces the biological function and homeostasis of mitochondria, promote cellular damage, and lead to cell death [144]. High level of oxidative molecules have been detected in plasma, and the exhaled breath and saliva UC patients, and UC pathogenesis is positively correlated with oxidative stress [145].

ROS include superoxide anions, free radicals, hydroxyl radicals, hydrogen peroxide, and singlet oxygen, which are by-products of normal oxygen metabolism [75]. Low-level ROS are necessary for certain physiological processes, like the phosphorylation of proteins, activation of transcription factors, cell differentiation, apoptosis, inflammatory response, and other physiological processes [146]. However, excessive ROS production harms cellular components, resulting in apoptosis and inflammation [147]. ROS in colonic tissues are derived from mitochondria and NADPH oxidase in epithelial and phagocytic cells [148]. Notably, basal levels of ROS in intestinal epithelial cells differ at different sites [149]. Basal ROS and antioxidant enzyme activities were higher in colonic epithelial cells than in small intestinal epithelial cells, which may be associated with the increased 8-oxo-deoxyguanosine DNA adducts levels in colonic epithelial cells during oxidative stress [150]. Differences in ROS production in epithelial cells may affect DNA damage, the levels of oxidized proteins, and lipids, resulting in increased susceptibility of the colon to oxidative stress [151].

IBD is commonly found in the digestive system. It is harmful to the physical and mental health of patients and may increase the risk of CRC [152]. A study on animal models of IBD showed significantly increased ROS production and further infiltration of macrophages and neutrophils in lamina propria of diseased colon and mice [153]. The evidence suggests that oxidative stress may be the main mechanisms for inflammatory response in the pathogenesis of IBD, evidenced by the significantly increased ROS production and reduced antioxidants levels in the diseased intestinal mucosa in animal models of IBD [154]. ROS can attack intestinal epithelial cell components, leading to colonic tissue damage, and stimulate leukocytes (mainly PMNs) to enhance the inflammatory response in IBD pathogenesis, further causing tissue damage [155]. Although lipid peroxidation is present in patients with IBD, it may originate from different sources depending on the type of IBD. Studies have shown that lipid peroxidation in CD is associated with mitochondrial superoxide dismutase activity, and lipid peroxidation products is connected with epithelial peroxidase catalase (CAT) activity in UC [156]. Additionally, lipid peroxidation products are mutagenic and carcinogenic [157].

Nuclear factor erythroid 2–related factor 2 (NRF2), a major player in the regulation of oxidative stress in cells, is involved in signaling pathways that maintain normal mitochondrial function, restore redox capacity, and inhibit inflammation development in several diseases [158]. Kikuchi et al. observed high levels of inflammation and fibrosis-related oxidative stress in NRF2^−/−^ KO mice [159]. The report showed that Oleuropein alleviated intestinal inflammation by reducing iNOS, COX2, TNF-α, and MCP-1 mRNA expression and activating the NRF2/HO-1 pathway in a DSS-induced IBD model [160]. In addition, Yang et al. found that syringic acid (CA) inhibited DSS-induced inflammation in mice through decreasing inflammatory mRNA expression, and the mRNA expression of NRF2 target genes SOD2, GCLM, GPX2, and HO-1 was increased in colonic tissues, suggesting that CA regulates NRF2-mediated oxidative stress to suppress inflammatory factors in intestinal inflammation [161,162]. In addition, the beneficial role of NRF2 in intestinal inflammation was investigated in NRF2^−/−^ KO mice that exhibited signs of severe colitis [153].

#### 3.1.3. Mitochondrial Dynamics-Inflammation-IBD

Mitochondria can autonomously translate cytoplasmic signs into functions compatible with cellular needs through remodeling cristae, fusion, and division [163]. The dynamic changes among mitochondrial fusion, fission, and biogenesis is essential for mitochondrial homeostasis [6]. Mitochondrial fusion allows damaged mitochondria to compensate for each other’s defects, resulting in the formation of new mitochondria [164]. By contrast, mitochondrial fission allows damaged mitochondria to be separated and transported to lysosomes for processing and healthy mitochondria to be reintegrated into the mitochondrial network [165]. Impairment of mitochondrial dynamics leads to abnormal ROS production and release, abnormal mtDNA production, and disturbances in energy metabolism, which stimulate inflammatory factors and inflammasomes and activate inflammation-related pathways [166]. According to the literature, an imbalance in mitochondrial status may underlie gastrointestinal dysfunction and lead to the expression of inflammatory factors, exacerbating intestinal inflammation [122].

A severe imbalance in mitochondrial dynamics was found in DSS- and dinitrobenzene sulfonic acid (DNBS)-induced mouse colitis models [167]. Changes in the mRNA levels of various proteins involved in mitochondrial dynamics related to colitis have been observed in mouse colonic tissue, suggesting that mitochondrial fusion and fission are involved in the development of colitis [168]. The mitochondrial fission inhibitor P110 has been reported to alleviate the severity of DSS and DNBS-induced colitis by increasing mitochondrial fission and fusion-related mRNA expression and phosphorylation of DROP1 [169]. Additionally, P110 alleviated DSS-induced epithelial cell energy disruption and mitochondrial debris production. Overall, the P110 treatment alleviated the inflammatory response by balancing the dysfunction of fission and fusion induced by DSS [170]. PGC-1α regulates energy metabolism and mitochondrial biosynthesis. Studies have reported that PGC-1α protein levels are significantly decreased in mice with colitis, and in humans with IBD [171]. In addition, mice without PGC1α in the intestinal epithelium exhibited more severe inflammation than wild-type mice, and these intestinal epithelial PGC1α knockout mice showed a reduced mitochondrial mass, increased ROS release, and reduced OXPHOS during the inflammatory process [134,170]. In total, mitochondrial homeostasis is essential for IBD development as shown Table 1.

#### 3.1.4. mtDNA-Induced Inflammation in IBD

mtDNA, the genetic material of mitochondria, is considered a pro-inflammatory DAMP with a pathogenic role in IBD [174]. mtDNA release into the cytoplasm and extracellular environment stimulates various immune responses, resulting in inflammation [175]. The patients with UC and CD have high plasma mtDNA levels than in those without IBD. These levels were correlated with clinical, blood, and endoscopic severity markers and disease activity [176]. In addition, mitochondrial damage and fecal mtDNA levels were significantly elevated in inflamed UC mucosa, which supports the release of mitochondrial DAMP from the intestinal mucosa as the major source [177]. In addition, plasma mtDNA levels were elevated during acute DSS colitis induction and were associated with severe colitis. Overall, mtDNA abnormalities were positively correlated with IBD development.

**Figure 4:**
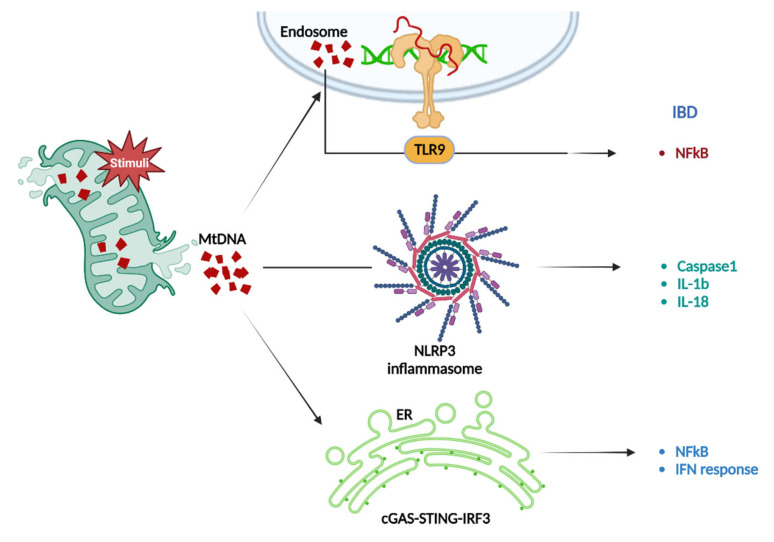
mtDNA mediated IBD through different pathways.

Studies have found that mtDNA stimulates Toll-like receptors (TLRs), cGAS, and inflammasomes, activating various inflammatory pathways as shown Figure 4. First, TLR9 is the recognition receptor for mtDNA, which is highly ex-pressed in the intestine, and TLR9 deficiency protects against colitis, indicating that mtDNA-TLR9 signaling is critical and targetable pathway for IBD treatment [177]. In addition, the NLRP3 inflammasome belongs to NLR family, and mtDNA plays a key role in NLRP3 inflammasomes activation, resulting in biologically active IL-1β production [178]. The literature has reported that mtDNA-bound NLRP3 inflammasome and IL-1β expression levels are higher in diseased intestinal tissues than in tissues from healthy groups [179]. Furthermore, WT mice that received DSS and mtDNA injections had increased severe colitis and high levels of IL-1β [180]. This evidence suggests that mtDNA leakage from dysfunctional mitochondria may exacerbate IBD inflammation by activating the NLRP3 inflammasome [181]. STING was originally regarded as a critical immune molecule, and there is growing evidence that STING pathway activation leads to tissue inflammation and injury [182]. mtDNA promotes inflammatory responses by activating the cGAS/STING/interferon regulatory factor 3 (IRF3) pathway [183]. According to the literature, mtDNA in damaged intestinal epithelial cells translocates to macrophages during CD and activates the STING pathway to elicit an inflammatory response [184]. Notably, STING deficiency ameliorated mtDNA-induced inflammatory activation. Overall, mtDAMPs released by mitochondrial damage has an essential role in the onset and inflammatory responses in IBD. The potential role of drugs, and targets, to protect mitochondrial function in the prevention and treatment of IBD should be investigated.

Mitochondrial stress can lead to mtDNA release. First, mtDNA can activate a serious of pro-inflammatory signaling pathways in IBD via endosomal-localized TLR9. Second, mtDNA-dependent inflammatory vesicle activity leads to caspase-1-dependent maturation or pro-inflammatory IL-1 and IL-8. Finally, cGAS recognizes mtDNA in the cytoplasmic lysate and activates STING localized to the endoplasmic reticulum (ER), thereby triggering an interferon response.

### 3.2. Mitochondria and Inflammation in CRC

#### 3.2.1. Mitophagy and Inflammation in CRC

Mitochondria are important organelles for cellular growth, development, metabolism and provide energy and substrates for cellular activities [185]. When cells are in a hostile environment, they remove unwanted or damaged mitochondria through autophagy, reducing their load and replenishing nutrients [186]. Mitophagy plays an important role in immune response, cell survival, resistance to environmental stress, aging, and apoptosis [187]. Abnormalities in mitophagy often lead to increased inflammation, and prolonged inflammatory responses can increase the incidence of tumors [188]. Due to lifestyle changes, the incidence and mortality rate of CRC is increasing annually, becoming one of the most common malignancies. Mitophagy plays an critical role in the development of CRC [112]. 

PINK1 is a mitochondrial kinase that regulates mitophagy and promotes cellular survival [189]. PINK1 deficiency leads to abnormal mitophagy, increases the accumulation of mtDNA mutations, and activates STING-mediated inflammatory pathways [69]. Long-term chronic inflammatory response increases the risk of malignant lesions [190]. The study shown that CRC patients has lower PINK1 level than normal human in colon tissues [191]. PINK1 overexpression promotes mitophagy, reduces glycolysis, and increases mitochondrial respiration, possibly by activating the p53 signaling pathway in mouse colon tumor cells [191]. Furthermore, in two mouse models of colitis-associated CRC, PINK1 disruption increased TNFα, IL-1β, IL-6, and IFN-β mRNA expression, and increased colon tumorigenesis, suggesting a role for PINK1 as a tumor inhibitor in CRC [191,192]. Parkin is an important regulator of mitochondrial homeostasis and plays critical role in biogenesis, fusion/fission, mitochondrial DNA repair, and mitophagy [69]. Parkin protein expression levels were lower in colon tumors than in normal tissues [193]. However, these levels were increased in patients with advanced colorectal cancer and were independent predictors of increased survival [194]. This evidence determined that Parkin may play a protective role in tumors. Tanshinone IIA may enhance CRC apoptosis in a mitochondria-dependent manner by inhibiting Parkin-mediated mitophagy [195].

HSP60 is responsible for maintaining the homeostasis of mitochondrial proteins and plays an essential role in maintaining mitochondrial function [196]. The high expression of HSP60 in tumors than that in healthy cells suggests that it may favor tumor growth [197]. The literature has reported that HSP60 knockdown inhibits cell proliferation by disrupting mitochondrial homeostasis in CRC [198]. HSP60 can bind to TOLL-like receptor 4 (TLR4) and activate the classical inflammatory pathways, NF-kB and JNK, inducing the release of TNFα [199]. Tumor necrosis factor type 1 receptor-associated protein (TRAP1), an HSP90 homolog, is mainly localized in mitochondria [200]. Patients with chronic UC have a high risk of developing CRC. TRAP1 was found to be highly expressed in chronic UC-associated CRC progression and was only positively correlated with the degree of inflammation in CRC tissue [201]. TRAP1 can promote tumor metastasis by inducing mitochondrial fission [202]. TRAP1 is also involved in aerobic glycolytic conversion, reducing OXPHOS and improving glucose utilization [203]. TRAP1 has complex effects on healthy and tumor cells; therefore, deepening the understanding of its role in different factors’ presence of TRAP1 and its role in the transition from the healthy to the tumor state is crucial to developing new CRC therapies [204].

#### 3.2.2. ROS and Inflammation in CRC

CRC is the third most common malignancy worldwide and the fourth deadliest recurrent metastatic tumor. Inflammation is one of the major factors contributing to CRC development [112]. There is important crosstalk between ROS, inflammation, and cancer. Increased ROS levels activate redox transcription factors, which increase the inflammatory genes expression, and tumor initiation requires only the presence of inflammatory cytokines [205]. Inflammatory features contribute to tumor progression and metastasis in CRC. Carcinogenesis is usually slow because of the development of chronic inflammation that promotes its occurrence and progression [188]. This often takes decades from tumorigenesis to diagnosis, and its development depends on several mechanisms. The process of the mutation and transformation of normal tissue cells into cancer cells stimulated by inflammation can be triggered at an early stage by the accumulation of free radicals, which are subsequently involved in cancer development [188]. Free radicals produced by enterococci in the colon may directly lead to mutations in coelomic DNA, leading to colon cancer [206]. Oxidative stress may provide an opportunity for colon cancer development though leading to genetic instability, specific gene alterations, and aberrant methylation, which induces intestinal epithelial cell damage and the development of inflammatory responses [207]. Excess ROS genes can induce the onset of oxidative stress in tissues or cells, further inducing oxidative DNA damage and ultimately activating intrinsic mitochondria-mediated inflammatory disease and extrinsic death receptor-mediated apoptotic pathways, promoting the development and progression of CRC [208]. Patients with IBD have a significantly high risk of developing cancer than individuals without IBD. Studies have shown that the risk of CRC increases with the duration of the clinical symptoms of IBD and the severity of the inflammatory response [209]. CRC complicated by IBD usually begins with mild atypical hyperplasia and progresses to indeterminate atypical hyperplasia, highly atypical hyperplasia, and invasive bowel cancer [210]. ROS-induced DNA damage and inflammation may be major factors in the development of colorectal cancer in patients with UC [211].

ROS play multiple roles in normal cells, and maintaining normal ROS levels is important for maintaining cellular functions and regulating cellular immune responses [212]. However, ROS level has two effects on the cell, depending on the concentration of ROS, the origin of the cancer cells, and the signaling pathways activated during tumor progression [151]. In general, moderate ROS levels lead to cellular damage, DNA mutations, and inflammation, which contribute to cancer development and progression [205]. A related study showed that ROS levels were higher in CRC cells compared with normal cells and ROS promoted cancer cell proliferation. Increased serum ROS levels were detected in 76 patients with CRC [213]. Zengjun et al. showed that abnormal levels of ROS promote CRC progression through the activation of chemokine CXCL14-related pathways [214]. ROS can silence the tumor suppressor RUNX3 through epigenetic regulation, promoting CRC progression [215]. CRC is closely associated with inflammation resulting from oxidative stress; therefore, the assessment of oxidative stress and the administration of antioxidants are important for CRC treatment and prevention [213].

NRF2 plays two roles in tumor development: it protects normal cells from transforming into cancer cells and promotes cancer cell proliferation [216]. In a colitis-associated cancer model induced by Kho r et al., NRF2 knockout mice showed increased expression of inflammatory factors in vivo and increased cancer susceptibility [217]. This finding suggests that NRF2 prevents inflammation and decreases cancer susceptibility [218]. Kang et al. used lignocaine to suppress the growth of cancer cell by inducing apoptosis, and its mechanism of action increased NRF2 transcription, the interaction between NRF2 and P53, and the expression of antioxidant enzymes and apoptosis-related proteins [219]. Siying et al. investigated the relationship between rapamycin and garlic and found that the combination of rapamycin and garlic-derived S-allylthiocysteine inhibited murine tumor growth by activating the NRF2 signaling pathway [218,220]. However, in the advanced stages of CRC, high NRF2 expression promotes CRC progression. Lee et al. examined NRF2 expression in 30 rectal cancer tissues and found higher NRF2 protein expression than in normal tissues [221]. NRF2 activates the PI3K/AKT signaling pathway and accelerates the progression of HCC [222]. ROS-induced NRF2 overexpression promotes inflammation and tumor formation in colonic tissues [223]. Studies have reported higheNRF2 expression, reduced apoptosis, and uncontrolled proliferation in tissues exposed to inflammatory macrophages [223]. In this context, this promotes colonic inflammation, reduces apoptosis, promotes cell proliferation, and promotes tumor formation. Thus, increased NRF2 expression may increase the risk of CRC [221].

#### 3.2.3. Mitochondrial Dynamics and Inflammation in CRC

Mitochondrial dynamics, critical for the maintaining mitochondrial function and inflammatory response in CRC, are regulated by specialized proteins and lipids [224]. Dysregulation of mitochondrial dynamics is related with the development and progression of multiple human cancers, affecting cancer metastasis, cancer stem cell survival and drug resistance, suggesting that regarding mitochondrial dynamics as a prospective therapeutic strategy [225].

According to related reports, NF-κB signaling participates in mitochondrial dysfunction in CRC [226]. Silencing COX-1 causes to the depolarization of the mitochondrial membrane potential, increased intracellular ROS production, and cysteine-dependent mitochondrial apoptosis [227]. In addition, the depletion of COX-1 inhibited NF-κB phosphorylation, resulting in the inhibition of anti-apoptotic Bcl-2 and enhanced expression of the pro-apoptotic Bax protein [228].

PGC-1α is extremely expressed in mitochondria and critical in regulating mitochondrial biogenesis [229]. PGC-1α participates in proliferation, the progression of cancer, invasion, and some metabolic pathways [230]. According to the literature, hypoxia increases PGC-1α expression and inhibits ROS production, and the upregulation of PGC-1α is relevant to proliferation, enhanced motility, and spheroid formation in CRC cells [231]. In addition, PGC-1α inhibits ER stress and apoptosis by decreasing antioxidant enzyme activity, cell survival, and the oxygen consumption ratio [232].

MFN2 is a mitochondrial outer membrane protein that is critical for mitochondrial homeostasis regulation by regulating mitochondrial fusion [233]. In addition, MFN2, a cell proliferation inhibitor, reportedly inhibits CRC cells proliferation by producing cell cycle arrest at the G2/M phase and increasing the levels of active caspase-3 [234]. Similarly, a dynamic balance between mitochondrial fission and fusion is crucial to maintain cellular homeostasis [235]. Evidence shows that Drp1 is related to the progression of cell cycle, cell proliferation, invasion, and apoptosis in different cancer cells [236]. For example, Drop1 depletion inhibits proliferation and promotes apoptosis in human colon cancer cells by increasing cytochrome C release and decreasing the mitochondrial membrane potential [237]. In addition, NaBt causes to cell cycle arrest and apoptosis in human CRC cells by regulating Drp1 levels to regulate mitochondrial fusion and division [238]. Overall, homeostasis of mitochondrial is inextricably linked to CRC onset and development as shown Table 2.

#### 3.2.4. mtDNA-Mediated Inflammation in CRC

mtDNA, an important DAMP released by damaged mitochondria after infection and stress, induces inflammatory responses through multiple pattern recognition receptors [239]; its mutation and dysfunction can be used to assess risk factors for cancer development. The low mtDNA copy number is connected with negative prognosis in CRC. In addition, according to other reports, the mtDNA copy number was lower in CRC tissues than in the corresponding non-cancerous colorectal tissues and significantly correlated with lymph node metastasis [240]. Overall, mitochondrial stability is critical for the regulation of CRC development.

TFAM is a multifunctional DNA-binding protein that is critical for transcriptional action and mtDNA organization and is required for the maintenance of mtDNA. [241]. TFAM knockdown increases the susceptibility to azoxymethane/DSS-induced CAC in mice, and TFAM overexpression prevents intestinal inflammation and colitis-related tumorigenesis in mice [242]. Colonic inflammation caused by injury and pathogens can increase susceptibility to CRC [243]. mtDNA released as a result of mitochondrial damage can act as act as an inducer of NLRP3 inflammasome. The mechanism of the NLRP3 inflammasome in CRC tumorigenesis suggests that variations in the NLRP3 gene are associated with CRC susceptibility [244]. Additionally, NLRP3 inflammasome knockout mice showed increased hepatic CRC metastasis [245]. STING is an innate immune sensor for cytosolic DNA essential for mediating mtDNA-triggered inflammation [246]. According to the literature, patients with high STING expression appear to have early-stage cancer with promoted infiltration of CD8^+^ T cells into the tumor [247]. Compared with the CRC patient with low STING expression, the patient who had high expression of STING had longer overall survival and recurrence-free survival [247]. These results suggest that [246,247]. Moreover, intratumoral injection of STING agonists in MC38 colon tumors brought about significant suppression of tumor progress and increased infiltration of CD8^+^ T cells within the tumor [248]. Overall, STING may be a prospective treatment target for enhancing the anti-cancer immune response in CRC.

## 4. Perspectives

Mitochondrial homeostasis is strongly related with the progression of IBD and CRC. Mitochondria maintain a normal immune response by regulating ROS production, apoptosis, mitochondrial dynamics, and mitophagy. Simultaneously, mitochondria regulate the development and progression of inflammation. The release of mtDNA as a result of mitochondrial damage stimulates TLRs, cGAS, and STING, activating various inflammatory pathways and exacerbating tumor progression. Prolonged inflammatory stimulation of IBD increases the appearance and progression of malignant lesions (CRC). As highlighted in this review, mitochondria-mediated inflammatory signaling plays a essential role in the development and progression of IBD and CRC. Overall, mitochondria are the major regulators of inflammation, and the goal is to target mitochondrial function as a means of controlling IBD and CRC triggered by inflammatory responses. Understanding the role and mechanisms of mitochondria in the inflammatory response will provide new therapeutic targets which could be used for the treatment of many serious inflammatory illnesses and cancers caused by inflammation.

## Figures and Tables

**Table 1:** The role of mitochondrial homeostasis related proteins in IBD.

Gene	Effect	Reference
ATG7	ATG7 deletion in intestinal anti-gen-presenting cells causes mitochondrial dysfunction and oxidative stress, and subsequently exacerbate inflammatory Th17 response	[126]
ATG16L1	ATG16L1 deficiency induces abundant ROS production and mitophagy defect, which enhanced colitis in mouse model via increasing Il-1β and TNFα mRNA expression	[129]
HSF2	HSF2 promotes mitophagy by reducing ROS and alleviates mucosal inflammation through inhibiting NLRP3 inflammasome via PARL/PINK1/PARKIN pathway	[133]
NIX	NIX deficient induces mitophagy defect and subsequently inflammatory features and loss of mucosal integrity in mouse experiments	[134]
PHB	PHB deletion in intestinal epithelial cells causes mitochondrial dysfunction and mitophagy defect, resulting in enhanced TNF-α, IL-1β, and reduced IL-10 mRNA expression, exacerbating the inflammatory response	[137]
IRGM1	Knockdown IRGM1 could affect inflammatory response by regulating mitophagy in intestinal epithelial cells and Paneth cells	[128]
NRF2	Oleuropein treatment alleviates intestinal inflammation by activating NRF2/HO-1 pathway and reducing iNOS, TNF-α and MCP-1 mRNA expression in DSS-induced IBD modle	[172]
PGC1α	PGC1α deficiency in intestinal epithelium shows more severe inflammation, reduces mitochondrial mass, and increases ROS production	[173]

**Table 2:** The role of mitochondrial homeostasis related proteins in CRC.

Gene	Effect	Reference
PINK1	PINK1 overexpression enhances mitophagy, reduces glycolysis, and increases mitochondrial respiration in mouse colon tumor cells. In contract, PINK1 deletion increases pro-inflammatory mRNA expression and colon tumorigenesis.	[191]
HSP60	HSP60 knockdown inhibits cell proliferation through disrupting mitochondrial homeostasis in CRC. Also, HSP60 can combine with TRAP1 and activates inflammatory pathways, subsequently release the TNFα	[198]
NRF2	ROS-induced NRF2 overexpression exacerbate inflammatory response and tumor formation in colonic tissues. In addition, high NRF2 level alleviated apoptosis and uncontrolled proliferation in tissue exposed to inflammatory macrophages	[217]
PGC-1α	Hypoxia induces PGC-1α expression, which is associated with enhanced motility, proliferation, and spheroid formation in CRC cells.	[231]
MFN2	MFN2 inhibits CRC cells proliferation through promoting cell-cycle arrest at G2/M phase and increasing active caspase-3 levels	[234]
DROP1	Slicing DROP1 inhibits human colon cancer cells and promotes its apoptosis through cytochrome C release and decreasing mitochondrial membrane potential	[237]

## Data Availability

Not applicable.

## Abbreviations

IBD	inflammatory bowel disease
CRC	colorectal cancer disease
mtDNA	mitochondrial DNA
CD	Crohn’s disease
DSS	dextran sodium sulfate
HSP	heat shock protein
IMM	the inner mitochondrial membrane
Irgm1	Immune-related GTPase family M protein
COX	cytochrome c oxidase
DAMP	damage-associated molecular pattern
Drp1	dynamin-related protein 1
DNBS	dinitrobenzene sulfonic acid
OMM	outer mitochondrial membrane
Opa1	optic atrophy 1
ROS	reactive oxygen species
TNF-a	tumour necrosis factor alpha
IFN-γ	interferon-γ
RNS	reactive nitrogen species
MID49/51	mitochondrial dynamics proteins of 49 and 51 kDa
FIS1	fission protein 1
MFF	mitochondrial fission factor
DRP1	dynamin-related protein 1
GED	GTPase effector structural domain
Parkin	E3 Ubiquitin-Protein Ligase
Pink1	PTEN Induced Kinase 1
NDP52	nuclear dot protein 52
OPTN	optineurin
USP30	ubiquitin-specific protease 30
TAX1BP1	TAX1 bindingprotein 1
NBR1	neighborof BRCA1 gene protein
LIR	LC3-interacting region
UBD	ubiquitin-binding domain
TBK1	TANK-binding kinase 1
UC	ulcerative colitis
ULK1	Unc-51-like kinase 1
OXPHOS	oxidative phosphorylation
ATP	adenosine triphosphate
MRC	mitochondrial respiratory chain
mtROS	Mitochondrial ROS
IRGM	immunity-related GTPase M
LRRK2	leucine-rich repeat kinase 2
Phb1	prohibitin 1
NF-κB	nuclear factor-kappa B
NRF2	nuclear factor erythroid 2–related factor 2
TLRs	Toll-like receptors
IRF3	interferon regulatory factor 3
TLR4	TOLL-like receptor 4
TRAP1	Tumor necrosis factor type 1
